# Relationship among Medical Student Resilience, Educational Environment and Quality of Life

**DOI:** 10.1371/journal.pone.0131535

**Published:** 2015-06-29

**Authors:** Patricia Tempski, Itamar S. Santos, Fernanda B. Mayer, Sylvia C. Enns, Bruno Perotta, Helena B. M. S. Paro, Silmar Gannam, Munique Peleias, Vera Lucia Garcia, Sergio Baldassin, Katia B. Guimaraes, Nilson R. Silva, Emirene M. T. Navarro da Cruz, Luis F. Tofoli, Paulo S. P. Silveira, Milton A. Martins

**Affiliations:** 1 Center for Development of Medical Education, School of Medicine of the University of São Paulo, Sao Paulo, Brazil; 2 Evangelical Medical School of Parana, Curitiba, Brazil; 3 Department of Medicine, School of Medicine of the University of Sao Paulo, Sao Paulo, Brazil; 4 Department of Obstetrics and Gynecology, Federal University of Uberlandia, Uberlandia, Minas Gerais, Brazil; 5 State University of São Paulo, Botucatu, Brazil; 6 ABC Foundation Medical School, Santo Andre, Brazil; 7 School of Medicine of Marilia, Marilia, Brazil; 8 School of Medicine of Rio Preto, Rio Preto, Brazil; 9 Department of Psychiatry, School of Medicine, State University of Campinas, Campinas, Brazil; 10 Department of Pathology, School of Medicine of the University of Sao Paulo, Sao Paulo, Brazil; Sickle Cell Unit, JAMAICA

## Abstract

**Context:**

Resilience is a capacity to face and overcome adversities, with personal transformation and growth. In medical education, it is critical to understand the determinants of a positive, developmental reaction in the face of stressful, emotionally demanding situations. We studied the association among resilience, quality of life (QoL) and educational environment perceptions in medical students.

**Methods:**

We evaluated data from a random sample of 1,350 medical students from 22 Brazilian medical schools. Information from participants included the Wagnild and Young’s resilience scale (RS-14), the Dundee Ready Educational Environment Measure (DREEM), the World Health Organization Quality of Life questionnaire – short form (WHOQOL-BREF), the Beck Depression Inventory (BDI) and the State-Trait Anxiety Inventory (STAI).

**Results:**

Full multiple linear regression models were adjusted for sex, age, year of medical course, presence of a BDI score ≥ 14 and STAI state or anxiety scores ≥ 50. Compared to those with very high resilience levels, individuals with very low resilience had worse QoL, measured by overall (β=-0.89; 95% confidence interval =-1.21 to -0.56) and medical-school related (β=-0.85; 95%CI=-1.25 to -0.45) QoL scores, environment (β=-6.48; 95%CI=-10.01 to -2.95), psychological (β=-22.89; 95%CI=-25.70 to -20.07), social relationships (β=-14.28; 95%CI=-19.07 to -9.49), and physical health (β=-10.74; 95%CI=-14.07 to -7.42) WHOQOL-BREF domain scores. They also had a worse educational environment perception, measured by global DREEM score (β=-31.42; 95%CI=-37.86 to -24.98), learning (β=-7.32; 95%CI=-9.23 to -5.41), teachers (β=-5.37; 95%CI=-7.16 to -3.58), academic self-perception (β=-7.33; 95%CI=-8.53 to -6.12), atmosphere (β=-8.29; 95%CI=-10.13 to -6.44) and social self-perception (β=-3.12; 95%CI=-4.11 to -2.12) DREEM domain scores. We also observed a dose-response pattern across resilience level groups for most measurements.

**Conclusions:**

Medical students with higher resilience levels had a better quality of life and a better perception of educational environment. Developing resilience may become an important strategy to minimize emotional distress and enhance medical training.

## Introduction

The term resilience has been used to refer to a person’s capacity to resist adversity without developing physical, psychological or social disabilities [1–8]. The resilience concept is still in debate. It has been considered as a trait, a set of personal characteristics, a process and/or a system. The concept of resilience as a trait implies that some people are naturally more resilient and capable to deal with adversities. As a set of personal characteristics, resilience encompasses confidence (self-efficacy), coordination (planning), control, composure (low anxiety) and commitment (persistence), that can facilitate persons moving on in a positive way from negative, traumatic or stressful experiences [1–6, 9–11]. In recent years, resilience has been considered as a process, where an individual, to be considered resilient, must have those personal characteristics tested in an objective or subjective adversity [2,9–12]. As a system, resilience is defined as the result of the interaction among the individual, his/her social support environment and the adversity, including his/her subject values, cultural, social and ethical influences [8,11–13].

It has been suggested that resilience influences medical student`s learning and medical professionalism [6–8]. The association between resilience and quality of life (QoL) has been studied in two directionalities, one assumes that resilient students have a better perception of quality of life either in a positive or negative educational environment [14–15], the second one considers interventions and a positive educational environment as factors to improve resilience in all medical students and increase their QoL and educational environment perceptions [12, 14–18].

Using this framework, we evaluated the association among resilience levels, QoL and educational environment perceptions in a multicenter random sample of Brazilian medical students. Our hypothesis was that there is a positive association among resilience, QoL and educational environment perceptions.

## Methods

### Study design

VERAS study (translated to English as “Students’ and Residents’ life in health professions”) is a multicenter study involving 22 Brazilian medical schools [19]. Schools participating in VERAS were geographically distributed across the country, with a diverse legal status and location (13 public and 9 private; 13 in state capital cities and 9 in other cities). Medical schools were from all geographic regions of Brazil, and were selected by convenience, i.e., if they had a research group willing to participate in the study. Data collection was performed from August 2011 to August 2012. The research protocol was approved by the Ethics Comittee of the School of Medicine of the University of Sao Paulo (Comitê de Ética em Pesquisa da Faculdade de Medicina da Universidade de São Paulo) and all medical schools included in the study (Universidade Federal do Rio de Janeiro, Universidade Federal de Ciências da Saúde de Porto Alegre, Universidade Estadual do Piauí, Faculdade de Medicina de Petrópolis, Faculdade de Ciências Médicas da Paraíba, Pontifícia Universidade Católica de São Paulo, Universidade Federal do Ceará, Universidade Federal de Goias, Universidade Federal de Mato Grosso do Sul, Escola Baiana de Medicina e Saúde Pública, Faculdade de Medicina de Marília, Faculdade de Medicina de São José do Rio Preto, Faculdade de Ciências Médicas da Paraíba, Faculdade Evangélica do Paraná, Faculdade de Medicina do ABC, Fundação Universidade Federal de Rondônia, Pontifícia UniversidadeCatólica do Rio Grande do Sul, Universidade Federal do Tocantins, Universidade Federal de Uberlândia, Universidade Estadual Paulista Júlio de Mesquita Filho, Centro Universitário Serra dos Orgãos, Universidade de Fortaleza and Universidade de Passo Fundo). All participants provided informed consent through electronic signature.

### Study sample

We randomly selected 60 students (5 males and 5 females per program year) from each medical school. This random sample was performed in clusters by gender and year of medical program, using a computer-generated list of random numbers. Students had a ten-day period to answer the survey online. In the case of no response, another student was randomly selected from the same cluster. Students received feedback on their scores once they responded to all questionnaires. All participants had the opportunity to contact the researchers for guidance or emotional support.

### Resilience assessment

Wagnild and Yong’s resilience scale is a short form measure of resilience, consisting of 14 items clustered in 5 domains: self-reliance, meaning, equanimity, perseverance and existential aloneness. Scores vary from 14 to 98, and higher scores indicate more resilience [5,20]. This questionnaire was translated and validated to Brazilian Portuguese [21].

### Quality of life assessment

We assessed students’ quality of life both as a global self-assessment and using a validated questionnaire, the World Health Organization Quality of Life Assessment (WHOQOL-BREF). The QoL self-assessment consisted of two questions to evaluate students' perception regarding their overall QoL and QoL related to medical school (MSQoL) on a scale from 0 to 10. The items were (1) rate your overall quality of life; (2) rate your quality of life in medical school. WHOQOL-BREF consists of 26 items clustered in four domains: environment, psychological, social relationships and physical health. Points within each domain are linearly transformed to a score from 0 to 100, and higher scores represent better QoL [22]. This questionnaire was translated and validated to Brazilian Portuguese [23].

### Perception of educational environment assessment

The Dundee Ready Educational Environment Measure (DREEM) is a questionnaire aimed to evaluate the educational environment. Its 50 items assess five domains: learning (scored 0 to 48), teachers (scored 0 to 44), academic self-perception (scored 0 to 32), atmosphere (scored 0 to 48) and social self-perception (scored 0 to 28). Global scores vary from 0 to 200, and higher scores mean a more positive perception about the educational environment [24]. This questionnaire was translated and validated to Brazilian Portuguese [25].

### Other assessments

To assess depression symptoms, we used the Beck Depression Inventory (BDI). This is a 21-item questionnaire and scores vary from 0 to 63, with higher scores pointing to either more numerous or more severe depressive symptoms[26]. Anxiety symptoms were assessed using the State Trait Anxiety Inventory (STAI), a two-component scale, with 20 items each, that evaluates two dimensions of anxiety: state and trait anxiety. Total state and total trait anxiety scores vary from 20 to 80 points [27]. Both questionnaires were translated and validated to Brazilian Portuguese [28].

The results of the reliability analyses performed using the Cronbach’s α coefficient demonstrated that the data were highly reliable, with α values between 0.70 and 0.94 for all domains of the questionnaires, except for the social domain of WHOQOL-BREF, that was 0.66 (data not shown).

### Study variables

Year of medical training was stratified in three levels of two years each, according to the most widely used classification in Brazilian schools: basic sciences (1^st^ and 2^nd^ years), clinical sciences (3^rd^ and 4^th^ years) and clerkships (5^th^ and 6^th^ years). Resilience levels were defined according to Wagnild and Young´s resilience scale global scores as very low (14 to 56 points), low (57 to 64 points), moderately low (65 to 73 points), moderately high (74 to 81 points), high (82 to 90 points) and very high (91 to 98 points) [5]. Perception of educational environment was categorized according to DREEM global scores as poor (0 to 50 points), with problems (51 to 100 points), positive (101 to 150 points) or excellent (151 to 200 points) [25]. The presence of depressive symptoms was defined according to BDI score as no depression (0 to 9 points), mild (10 to 17 points), moderate (18 to 29 points) or severe (30 to 63 points) depression [28]. Marked anxiety symptoms were defined according to STAI, as a score from 50 to 80 points in either anxiety state or anxiety trait scores [28,29].

### Statistical analysis

Sample size was defined to detect an effect size of 0.165 between two groups with the same size, with 80% power at a 0.05 significance level. We increased the sample to account for 30% of losses. We calculated a sample size of 1,152 students (576 men and 576 women). Categorical variables are presented as proportions and quantitative variables are shown as mean ± standard deviation (SD). Chi-squared and Fisher’s exact test were used whenever applicable. We compared WHOQOL, DREEM, BDI and STAI mean scores across resilience level groups using ANOVA with Tukey’s HSD test to adjust for multiple comparisons. For all comparisons, we used the very high resilience group as reference. We built multiple linear regression models to assess the association between resilience level and QoL measurements (self-assessment and WHOQOL domains) and the association between resilience level and educational environment measurements (DREEM global score and domains). We used, for each comparison, two models: (1) crude and (2) adjusted for sex, age, year of medical course, presence of depression and presence of marked anxiety symptoms. We also built *post-hoc* models adjusted and stratified by school legal status (public or private) and school location (state capital or other cities). All model results are presented as beta-coefficients and their respective 95% confidence interval. In all regression models we considered the very high resilience group as the reference group. We set significance level at 0.05. We conducted analyses using R software version 3.1.2.

## Results

**Fig 1:**
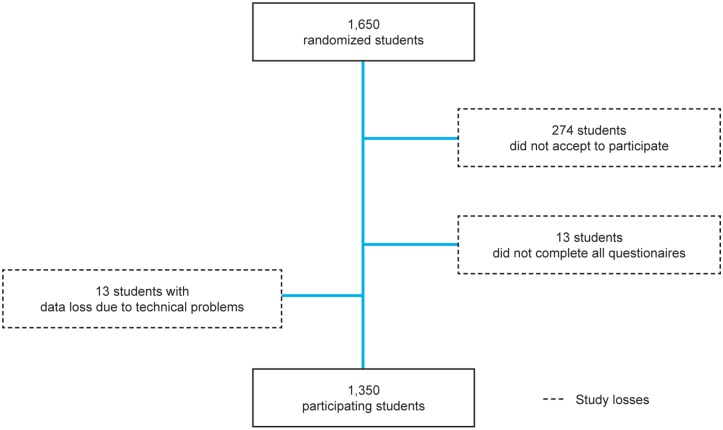
Study participants and losses flowchart.

Of 1,650 randomly selected students, 1,350 (81.8%) accepted to participate and completed the study (Fig 1). The main reason to refuse to participate in the study (16.6%) was lack of time. In our sample, 714 individuals (52.9%) were women, 459 (34.0%) were in the first and second years of medical course, 491 (36.4%) were in the third and fourth years and 400 (29.6%) in the last two years. Their ages ranged between 17 and 40 (22.8 ± 1.3 years old).

Sex (p = 0.60) and year of medical program (p = 0.46) were not significantly different across resilience levels groups. Students with very high resilience had lower BDI, state anxiety and trait anxiety scores when compared to all other resilience level groups (p<0.001 for all comparisons) (Table 1).

**Table 1:** Characteristics of study participants according to resilience level.

	Resilience levels and scores						
	Very High	High	Moderately High	Moderately Low	Low	Very Low	Total
	(91–98)	(82–90)	(74–81)	(65–73)	(57–64)	(14–56)	(14–98)
	N = 203	N = 435	N = 348	N = 188	N = 89	N = 87	N = 1,350
**Female sex**	100 (49.3%)	226 (52.0%)	188 (54%)	100 (53.2%)	47 (52.8%)	53 (60.9%)	714 (52.9%)
**Age** (Mean ± SD)	23.2 ± 3.3	22.9 ± 3.1	22.6 ± 2.9	22.6 ± 2.8	22.8 ± 2.8	22.1 ± 2.6	22.8 ± 3.0
**Year of medical course**							
1^st^ / 2^nd^ (basic sciences)	60 (29.6%)	149 (34.3%)	118 (33.9%)	68 (36.2%)	25 (28.1%)	39 (44.8%)	459 (34.0%)
3^rd^ / 4^th^ (clinical sciences)	76 (37.4%)	160 (36.8%)	133 (38.2%)	63 (33.5%)	33 (37.1%)	26 (29.9%)	491 (36.4%)
5^th^ / 6^th^ (clerkships)	67 (33.0%)	126 (29.0%)	97 (27.9%)	57 (30.3%)	31 (34.8%)	22 (25.3%)	400 (29.6%)
**Overall QoL** ^a^ (Mean ± SD)	8.4 ± 1.2	8.0 ± 1.2	7.8 ± 1.2	7.7 ± 1.2	7.3 ± 1.4	7.0 ± 1.3	7.9 ± 1.3
**Medical school-related QoL** (Mean ± SD)	7.1 ± 1.6	6.7 ± 1.5	6.6 ± 1.4	6.3 ± 1.4	5.7 ± 1.6	5.4 ± 1.7	6.5 ± 1.6
**WHOQOL scores**							
Environment (Mean ± SD)	69.1 ± 13.7	66.8 ± 13.6	63.0 ± 13.1	60.1 ± 12.2	57.2 ± 15.7	54.7 ± 14.1	63.8 ± 14.1
Psychological (Mean ± SD)	75.0 ± 11.4	67.0 ± 12.2	60.9 ± 12.4	53.9 ± 13.0	48.5 ± 12.6	37.9 ± 14.4	61.7 ± 15.7
Social relationships (Mean ± SD)	74.7 ± 17.5	66.3 ± 19.0	63.4 ± 18.6	58.2 ± 18.1	52.7 ± 18.6	47.3 ± 20.2	63.6 ± 19.9
Physical health (Mean ± SD)	74.1 ± 12.4	68.7 ± 13.8	64.5 ± 12.8	59.9 ± 12.8	55.9 ± 14.6	51.1 ± 15.4	65.2 ± 14.7
**Global DREEM score** ^b^ (Mean ± SD)	136.7 ± 26.1	126.6 ± 24.7	117.1 ± 24.1	108.6 ± 22.2	101.0 ± 22.9	94.7 ± 26.0	119.4 ± 27.1
Poor (0–50)	0	1 (0.2%)	0	0	3 (3.4%)	4 (4.6%)	8 (0.6%)
With problems (51–100)	22 (10.8%)	77 (17.7%)	84 (24.1%)	61 (32.4%)	35 (39.3%)	44 (50.6%)	323 (29.2%)
Positive (101–150)	119 (58.6%)	285 (65.5%)	237 (68.1%)	121 (64.4%)	51 (57.3%)	39 (44.8%)	852 (63.1%)
Excellent (151–200)	62 (30.5%)	72 (16.6%)	27 (7.8%)	6 (3.2%)	0	0	167 (12.4%)
**DREEM Domains scores**							
Learning (Mean ± SD)	31.5 ± 7.1	29.1 ± 7.5	27.0 ± 6.9	25.4 ± 6.9	24.2 ± 7.2	22.6 ± 6.7	27.6 ± 7.5
Teachers (Mean ± SD)	30.5 ± 7.1	28.9 ± 6.7	27.1 ± 6.7	26.0 ± 5.8	24.9 ± 6.0	23.8 ± 7.3	27.7 ± 6.9
Academic self-perception (Mean ± SD)	22.5 ± 4.5	20.2 ± 4.6	18.4 ± 4.3	16.8 ± 4.1	15.5 ± 4.5	13.9 ± 4.6	18.9 ± 5.0
Atmosphere (Mean ± SD)	34.0 ± 7.4	31.7 ± 6.6	29.2 ± 6.9	26.4 ± 6.9	24.2 ± 6.9	22.7 ± 8.0	29.6 ± 7.7
Social self-perception (Mean ± SD)	18.2 ± 4.4	16.7 ± 3.9	15.4 ± 3.9	13.9 ± 3.6	12.2 ± 4.2	11.7 ± 4.2	15.6 ± 4.4
**BDI** ^c^ **score** (Mean ± SD)	5.0 ± 4.3	7.3 ± 5.4	9.3 ± 5.6	11.7 ± 6.5	14.6 ± 6.9	20.6 ± 8.9	9.4 ± 7.0
No depressive symptoms (0–9)	173 (85.2%)	317 (72.9%)	193 (55.5%)	82 (43.6%)	20 (22.5%)	8 (9.2%)	793 (58.7%)
Mild depressive symptoms (10–17)	27 (13.3%)	20 (4.6%)	34 (9.8%)	32 (17.0%)	24 (27.0%)	43 (49.4%)	385(28.5%)
Moderate depressive symptoms (18–29)	3 (1.5%)	97 (22.3%)	121 (34.8%)	73 (38.8%)	42 (47.2%)	25 (28.7%)	156 (11.6%)
Severe depressive symptoms (30–63)	0	1 (0.2%)	0	1 (0.5%)	3 (3.4%)	11 (12.6%)	16 (1.2%)
**State anxiety score** (Mean ± SD)	36.7 ± 10.7	40.8 ± 10.6	44.4 ± 10.5	48.1 ± 10.2	49.7 ± 11.1	55.5 ± 11.2	43.7 ± 11.6
Marked anxiety symptoms (50–80)	26 (12.8%)	90 (20.7%)	107 (30.7%)	80 (42.6%)	44 (49.4%)	63 (72.4%)	410 (30.4%)
**Trait anxiety score** (Mean ± SD)	35.4 ± 9.1	41.9 ± 9.7	46.0 ± 9.3	51.5 ± 9.3	55.0 ± 9.1	62.2 ± 9.0	45.5 ± 11.7
Marked anxiety symptoms (50–80)	16 (7.9%)	95 (21.8%)	121 (34.8%)	103 (54.8%)	67 (75.3%)	78 (89.7%)	480 (35.6%)

^a^ QoL = Quality of Life;

^b^DREEM = Dundee Ready Educational Environment Measure;

^c^ BDI = Beck Depression Inventory.

Analyzing mean scores with adjustment for multiple comparisons using Tukey’s HSD, we found significant differences comparing students with very high resilience to others. Those with very high resilience had higher overall QoL than participants with very low, low, moderately low, moderately high (p<0.001 for all) and high (p = 0.013) resilience levels. Regarding medical school-related QoL, those with very high resilience had significantly higher scores than those with very low, low, moderately low (p<0.001 for all) and moderately high (p = 0.004) resilience levels. We also observed higher medical school-related QoL scores for those with very high resilience when compared to those with high resilience, with borderline significance after adjustment for multiple comparisons (p = 0.054). Using WHOQOL-BREF to assess quality of life, for the psychological, social relationships and physical health domains, we found higher scores in individuals with very high resilience compared to those with high, moderately high, moderately low, low and very low resilience groups (p<0.001 for all comparisons). For the environment WHOQOL domain, with the exception of a non-significant difference between the mean scores of the very high and the high resilience group (p = 0.33), those with very high resilience also had significantly higher scores compared to all other resilience groups (p<0.001 for all comparisons).

We found similar results analyzing the perception of educational environment according to DREEM scores, with adjustment for multiple comparisons. Mean DREEM global scores were higher in the very high resilience group compared to all other groups (p<0.001 for all comparisons). When we evaluated the results within each DREEM domain, we found significantly higher mean scores for the very high resilience group compared to individuals with moderately high, moderately low, low or very low resilience (p<0.001 for all comparisons) in all domains. Compared to individuals with high resilience, those with very high resilience still had significantly higher learning (p = 0.001), atmosphere (p = 0.002), academic self-perception (p<0.001) and social self-perception (p<0.001) DREEM domain scores and a non-significant trend towards higher scores in teachers’ DREEM domain (p = 0.073).

The results of multiple linear regression models to evaluate the association between resilience level groups and the measurements of QoL (Table 2) and for the perception of educational environment (Table 3) show progressive lower beta-coefficient estimates for lower resilience groups, suggesting a dose-effect pattern for these associations. Most of these differences were not attenuated when models were adjusted for sex, age, year of medical school, the presence of a BDI score above or equal to 10 points or marked anxiety symptoms. We also ran *post-hoc* models adjusted and *post-hoc* models stratified by school legal status (public or private) and by school location (state capital or other cities). As expected, because of the smaller sample size in each group and the higher number of variables in the models, some confidence intervals lost statistical significance in these *post-hoc* models. Almost all beta-coefficient point estimates remained negative in these models and in all cases that beta-coefficients remained significant they were negative, suggesting this association is not mediated solely by these school characteristics nor occur exclusively in specific scenarios.

**Table 2:** Crude and adjusted beta-coefficients for the association between resilience levels and quality of life perception.

	Resilience level					
	Very High	High	Moderately High	Moderately Low	Low	Very Low
**Overall QoL**						
Crude	Ref (0.0)	-0.34 (-0.55 to -0.14)	-0.52 (-0.74 to -0.31)	-0.72 (-0.96 to -0.47)	-1.07 (-1.37 to -0.76)	-1.39 (-1.70 to -1.08)
Adjusted	Ref (0.0)	-0.27 (-0.47 to -0.07)	-0.35 (-0.56 to -0.14)	-0.41 (-0.66 to -0.16)	-0.64 (-0.95 to -0.32)	-0.89 (-1.21 to -0.56)
**Medical school-related QoL**						
Crude	Ref (0.0)	-0.36 (-0.61 to -0.11)	-0.48 (-0.74 to -0.22)	-0.79 (-1.09 to -0.50)	-1.31 (-1.69 to -0.94)	-1.66 (-2.04 to -1.29)
Adjusted	Ref (0.0)	-0.24 (-0.48 to 0.00)	-0.18 (-0.44 to 0.07)	-0.30 (-0.60 to 0.00)	-0.60 (-0.99 to -0.22)	-0.85 (-1.25 to -0.45)
**WHOQOL environment**						
Crude	Ref (0.0)	-2.32 (-4.56 to -0.07)	-6.16 (-8.50 to -3.83)	-8.98 (-11.66 to -6.30)	-11.89 (-15.25 to -8.52)	-14.38 (-17.77 to -10.99)
Adjusted	Ref (0.0)	-1.08 (-3.23 to 1.06)	-3.32 (-5.61 to -1.04)	-4.32 (-7.00 to -1.64)	-5.43 (-8.83 to -2.02)	-6.48 (-10.01 to -2.95)
**WHOQOL psychological**						
Crude	Ref (0.0)	-7.99 (-10.06 to -5.92)	-14.11 (-16.26 to -11.96)	-21.16 (-23.63 to -18.70)	-26.57 (-29.66 to -23.47)	-37.09 (-40.21 to -33.97)
Adjusted	Ref (0.0)	-5.79 (-7.50 to -4.08)	-8.84 (-10.66 to -7.03)	-12.82 (-14.96 to -10.68)	-14.74 (-17.45 to -12.02)	-22.89 (-25.70 to -20.07)
**WHOQOL social relationships**						
Crude	Ref (0.0)	-8.39 (-11.49 to -5.28)	-11.26 (-14.49 to -8.03)	-16.47 (-20.17 to -12.77)	-21.96 (-26.60 to -17.31)	-27.35 (-32.04 to -22.67)
Adjusted	Ref (0.0)	-6.44 (-9.35 to -3.53)	-6.42 (-9.52 to -3.33)	-8.70 (-12.34 to -5.06)	-10.63 (-15.25 to -6.01)	-14.28 (-19.07 to -9.49)
**WHOQOL physical health**						
Crude	Ref (0.0)	-5.43 (-7.66 to -3.20)	-9.59 (-11.91 to -7.28)	-14.21 (-16.86 to -11.55)	-18.20 (-21.54 to -14.87)	-22.95 (-26.31 to -19.59)
Adjusted	Ref (0.0)	-3.44 (-5.46 to -1.43)	-5.05 (-7.20 to -2.90)	-7.09 (-9.62 to -4.57)	-8.51 (-11.71 to -5.31)	-10.74 (-14.07 to -7.42)

Results presented as beta-coefficients (95%confidence intervals); Adjusted models are adjusted for sex, age, year of medical course, presence of a BDI score ≥ 10 points and presence of marked anxiety symptoms (state or trait). QoL = Quality of life.

**Table 3:** Crude and adjusted beta-coefficients for the association between resilience levels and educational environment perception.

	Resilience level					
DREEM	Very High	High	Moderately High	Moderately Low	Low	Very Low
**Global score**						
Crude	Ref (0.0)	-10.05 (-14.12 to -5.97)	-19.55 (-23.78 to -15.32)	-28.09 (-32.94 to -23.24)	-35.69 (-41.79 to -29.60)	-41.99 (-48.13 to -35.85)
Adjusted	Ref (0.0)	-8.68 (-12.59 to -4.77)	-15.76 (-19.92 to -11.6)	-21.47 (-26.36 to -16.58)	-25.76 (-31.97 to -19.56)	-31.42 (-37.86 to -24.98)
**Learning**						
Crude	Ref (0.0)	-2.37 (-3.56 to -1.18)	-4.51 (-5.75 to -3.27)	-6.07 (-7.49 to -4.66)	-7.31 (-9.09 to -5.53)	-8.89 (-10.69 to -7.10)
Adjusted	Ref (0.0)	-2.21 (-3.38 to -1.05)	-3.95 (-5.19 to -2.72)	-5.02 (-6.47 to -3.56)	-5.58 (-7.42 to -3.73)	-7.32 (-9.23 to -5.41)
**Teachers**						
Crude	Ref (0.0)	-1.53 (-2.64 to -0.43)	-3.36 (-4.51 to -2.21)	-4.44 (-5.76 to -3.12)	-5.57 (-7.23 to -3.91)	-6.64 (-8.31 to -4.97)
Adjusted	Ref (0.0)	-1.42 (-2.51 to -0.34)	-2.97 (-4.13 to -1.82)	-3.58 (-4.94 to -2.22)	-4.23 (-5.95 to -2.51)	-5.37 (-7.16 to -3.58)
**Academic self-perception**						
Crude	Ref (0.0)	-2.37 (-3.11 to -1.64)	-4.15 (-4.91 to -3.38)	-5.72 (-6.60 to -4.85)	-7.03 (-8.13 to -5.93)	-8.66 (-9.77 to -7.55)
Adjusted	Ref (0.0)	-2.17 (-2.90 to -1.45)	-3.66 (-4.43 to -2.88)	-4.91 (-5.82 to -4.00)	-5.88 (-7.03 to -4.72)	-7.33 (-8.53 to -6.12)
**Atmosphere**						
Crude	Ref (0.0)	-2.26 (-3.41 to -1.10)	-4.78 (-5.99 to -3.58)	-7.60 (-8.98 to -6.22)	-9.81 (-11.54 to -8.08)	-11.30 (-13.05 to -9.55)
Adjusted	Ref (0.0)	-1.85 (-2.97 to -0.73)	-3.69 (-4.88 to -2.49)	-5.74 (-7.14 to -4.33)	-7.02 (-8.80 to -5.24)	-8.29 (-10.13 to -6.44)
**Social self-perception**						
Crude	Ref (0.0)	-1.52 (-2.19 to -0.85)	-2.75 (-3.44 to -2.05)	-4.25 (-5.04 to -3.45)	-5.97 (-6.97 to -4.97)	-6.50 (-7.51 to -5.49)
Adjusted	Ref (0.0)	-1.01 (-1.62 to -0.41)	-1.49 (-2.13 to -0.85)	-2.23 (-2.99 to -1.48)	-3.06 (-4.02 to -2.10)	-3.12 (-4.11 to -2.12)

Results presented as beta-coefficients (95%confidence intervals); Adjusted models are adjusted for sex, age, year of medical course, presence of a BDI score ≥ 10 points and presence of marked anxiety symptoms (state or trait).

## Discussion

We found that, consistently with our initial hypothesis, medical students with lower resilience levels had more negative QoL and educational environment perceptions. A dose-effect pattern was observed across resilience level groups. These associations were independent of sex, year of medical school and depressive or anxiety symptoms.

Resilience may impact quality of life and mental health [5,30]. Our data confirm this relationship, since we observed that higher resilience levels were associated with a better perception of self-reported QoL and the domains of WHOQOL-BREF. World Health Organization defines QoL as “individuals’ perceptions of their position in life in the context of the culture and value systems in which they live and in relation to their goals, expectations, standards and concerns” [22]. This perception can be more positive or negative according to the meanings that each person attributes to his/her life experiences. In this way, attributing a positive value and meaning to life experiences, even those negatives, is a major characteristic of resilient people [31].

In our study, medical students gave significantly higher scores for their overall QoL than to their medical school-related QoL. In a previous study, medical students were interviewed using focus groups to better explore the factors related to increases and decreases of their QoL during medical school. Students reported that quality of teachers, curricula, healthy lifestyles related to eating habits, sleep and physical activity influenced their QoL. Lack of time due to medical school obligations had a major impact on their QoL. However, they considered that their difficulties, although resulting in worse QoL, were necessary and inherent to the process of becoming doctors [32].

We also observed a significant association between higher resilience scores and lower scores of anxiety and depression. In fact, some authors include low anxiety and optimism among the characteristics of resilient people [5–7]. Causal relationship for this association can be bidirectional. First, a person’s resilience may be a protective factor against the development of anxiety and/or depressive symptoms. Second, an anxious or depressed person may be less able to use his/her coping skills. These two possibilities point to the dynamic aspects of resilience: an individual can put in practice their resilience skills in some moments and/or situations and not in others [2,11,12]. However, in the regression models, when we controlled our results for high anxiety and depression scores, the associations between high levels of resilience and both QoL and education environment perceptions were not attenuated, suggesting that anxiety or depression levels did not have a significant influence on our findings.

The educational environment embraces several factors that contribute to learning, it is everything experienced or perceived by students and teachers [24,33,34]. We found that resilient students had a better perception of their educational environment. Among students with resilience scores high or very high 15.7% had a DREEM score corresponding to an educational environment poor or with problems. In contrast, in students with low or very low resilience levels, this proportion was 48.9%. This difference is probably related to the strategies to face adversities during medical course. This is consistent with the findings from a prospective study by Dyrbye et al that observed that medical students with a better perception of their educational environment had a lower risk of developing burnout [35].

Many stressors in medical school have been described. For some authors, medical school may be an educational environment with psychological toxicity, where many experiences overwhelm rather than develop students [36–39]. Degrees of suffering vary according to personal abilities and social support [16,36]. Haglund et al studied stressful events during clerkship, in third-year medical students. Interestingly, students who reported more traumatic events had more personal growth at the end of the academic year. In contrast, unprofessional behavior by residents and attending physicians had adverse effects on the students’ well-being [40]. Tedeschi and Calhoun observed, in psychology students, that exposure to traumatic events was associated to better personal development [41]. However, other studies have demonstrated that, in the face of adversities, maladaptive responses may also occur. Students may develop higher levels of cynicism and decreased empathy [19,42], resulting in unprofessional conducts, less altruistic professional values [14] and worse well-being perceptions [40]. Resilience has been proposed as a mediator between the occurrence of stressful events and personal maturing [14], which can result in a new adaptive state [43].

Aligned to this concept, the Association of American Medical Colleges (AACME) included resilience as one out of nine personal characteristic associated with a successful behavior during medical training [44,45].

Most studies on the development of resilience have not focused on healthy adult populations. Studies regarding medical students are even scarcer. In qualitative study, Nagji et al observed a positive impact of an optional theatre module on relaxation, social relationships and resilience of 18 first-year medical students [18]. Slavin found a decrease in depressive and anxiety symptoms after a set of curricular changes, which included a required resilience and mindfulness program, focused on energy management, stress reduction and other coping strategies [46]. Similar results were described by Zamirinejad et al, in a sample of 31 women with a BDI score of 19 or higher. Those authors found that those who participated in the psychotherapy group for resilience training had similar pre-intervention BDI scores and lower post-intervention scores compared to the non-intervention group [17].

Surprisingly, in our study the resilience levels were not significantly different when we compared medical students from different years of medical school. During medical school, there are many factors that may influence resilience of medical students, in both directions. There are many opportunities for the students to develop their resilience. However, educational strategies specifically designed to improve resilience are still rare in medical schools in Brazil and around the world.

To our knowledge, all previous studies concerning resilience in medical students were performed in convenience samples and/or included a single or few medical schools [14,15,40]. VERAS study protocol consisted in survey in a large, random sample, from 22 Brazilian schools located in different cities, in all Brazilian regions and had a high (81.8%) response rates [19]. Our study also has some limitations. The cross-sectional design does not allow inferences of causality. However, this design is still adequate to study associations and provide wide data to discussion. In addition, as our sample was restricted to Brazilian medical students, the generalization of the results to other populations should be made with caution.

In conclusion, we found that medical students with higher resilience levels had better quality of life and educational environment perceptions. Our data are consistent with the concept that resilience is a core competency for medical school admission process.
